# Improving sensitivity of XANES structural fit to the bridged metal–metal coordination

**DOI:** 10.1107/S1600577524002091

**Published:** 2024-03-26

**Authors:** S. V. Abrosimov, B. O. Protsenko, A. S. Mannaa, V. G. Vlasenko, S. A. Guda, I. A. Pankin, A. S. Burlov, Y. V. Koshchienko, A. A. Guda, A. V. Soldatov

**Affiliations:** a The Smart Materials Research Institute, Southern Federal University, Sladkova 178/24, 344090 Rostov-on-Don, Russian Federation; b Institute of Physics, Southern Federal University, Stachki Ave 194, 344090 Rostov-on-Don, Russian Federation; c Institute of Mathematics, Mechanics and Computer Science named after I. I. Vorovich, Milchakova 8a, 344090 Rostov-on-Don, Russian Federation; d Institute of Physical and Organic Chemistry, Stachki Ave 194/2, 344090 Rostov-on-Don, Russian Federation; ESRF – The European Synchrotron, France

**Keywords:** XANES, EXAFS, machine learning, vibrational spectroscopy, radial basis functions, improved hypercube sampling

## Abstract

Hard X-ray absorption spectroscopy is a valuable *in situ* probe for non-destructive diagnostics of metal sites. The low-energy interval of a spectrum (XANES) contains information about the metal oxidation state, ligand type, symmetry and distances in the first coordination shell but shows almost no dependency on the bridged metal–metal bond length. The higher-energy interval (EXAFS), on the contrary, is more sensitive to the coordination numbers and can decouple the contribution from distances in different coordination shells. Supervised machine-learning methods can combine information from different intervals of a spectrum; however, computational approaches for the near-edge region of the spectrum and higher energies are different. This work aims to keep all benefits of XANES and extend its sensitivity towards the interatomic distances in the first and second coordination shells. Using a binuclear bridged copper complex as a case study and cross-validation analysis as a quantitative tool it is shown that the first 170 eV above the edge are already sufficient to balance the contributions of Cu–O/N scattering and Cu–Cu scattering. As a more general outcome this work highlights the trivial but often overlooked importance of using ‘longer’ energy intervals of XANES for structural refinement and machine-learning predictions. The first 200 eV above the absorption edge still do not require parametrization of Debye–Waller damping and can be calculated within full multiple scattering or finite difference approximations with only moderately increased computational costs.

## Abstract 2

Machine-learning methodology improves the sensitivity of XANES spectra to metal–metal bond distances in a bridged configuration.

## 1. Introduction

X-ray absorption spectroscopy (XAS) is a unique tool for analysis of local atomic and electronic structure around metal ions. Thanks to the development of modern synchrotron radiation sources this method became popular in the field of *in situ* and *operando* studies for coordination complexes and active sites of catalysts under gas, temperature and light interaction. The X-ray absorption spectrum is sensitive to the charge state, ligand type and coordination geometry around the absorbing atom. Such sensitivity allows two types of tasks to be solved – classification of the ligand environment (oxidation state or coordination motif) via the fingerprint approach or model refinement via regression (when the structural model is fixed, and its structural parameters are refined). The latter procedure is analogous to the Rietveld refinement in X-ray diffraction and implemented in the software *Artemis* (Ravel & Newville, 2005*a* ▸) and *Viper* (Klementev, 2001 ▸) for the extended X-ray absorption fine structure (EXAFS) and *MXAN* (Benfatto & Della Longa, 2009 ▸), *FitIt* (Smolentsev & Soldatov, 2007 ▸) and *PyFitIt* (Martini *et al.*, 2020*a* ▸) for the X-ray absorption near-edge structure (XANES).

Along with high sensitivity to the local atomic and electronic structure, overlapping contributions from all neighbor atoms reduce the analytical power of XAS compared, for example, with nuclear magnetic resonance (NMR). Contributions from different structural parameters (interatomic distances, coordination numbers, ligand environments) overlap with each other in every point of the spectrum. Several methods exist that can address such signal superposition. The Fourier transform was historically the first approach that disentangled contributions from different distances in the spectrum. The wavelet transformation further allows disentangling scatterers with different atomic number located at the same distance. For the near-edge fine structure, researchers are applying machine-learning (ML) methods more and more often. Zheng *et al.* (2020 ▸) applied random forest models to differentiate between 21 structural motifs using all points of the XAS spectrum as input. Carbone *et al.* (2019 ▸) applied a convolutional neural network to classify the entire X-ray absorption spectrum in terms of tetrahedral, square pyramidal or octahedral coordination. In a subsequent work they demonstrated that graph-based neural networks can solve the opposite task and predict the O *K*-edge and N *K*-edge X-ray absorption near-edge structure spectra of molecules to quantitative accuracy (Carbone *et al.*, 2020 ▸). Rankine & Penfold (2022 ▸) utilized theoretical metal *K*-edge spectra for more than 200000 structures in The Materials Project (Jain *et al.*, 2013 ▸) and trained the deep neural network which predicts the XANES spectrum for any given structure. A comprehensive prediction of Si *K*-edge XANES spectra was performed based on an atom-centered symmetry function, smooth overlap of atomic positions, local many-body tensor representation, and spectral neighbor analysis potential (Hirai *et al.*, 2022 ▸). Successfully refining the quality of prediction and eliminating systematic differences between theory and experiment, such approaches may replace calculation software in the future. Nowadays, training ML algorithms on smaller but more representative datasets is more common. Frenkel and co-workers have applied neural networks to analyze coordination numbers in Pt nanoparticles (Timoshenko *et al.*, 2017 ▸, 2019 ▸) by using the near-edge region of the spectra.

However, ML is not a miracle and the sensitivity of the chosen spectral interval to the studied properties plays an even more crucial role than sophisticated numerical ML methods. The understanding of local atomic structure of Me–Me or Me–oxo–Me binuclear species is highly relevant for Me-exchanged zeolites – a large class of materials used in heterogeneous catalysis (Borfecchia *et al.*, 2018 ▸). For Cu-zeolites an understanding of the structural evolution of such binuclear copper sites or even changes in their nuclearity under different reaction conditions is hotly debated in the literature. It is believed that both geometry and nuclearity of such species might determine the catalytic activity or even degradation rate of the catalysts (Vanelderen *et al.*, 2013 ▸). For such dimeric sites in zeolites, EXAFS spectroscopy suffers from weak and broadened Me–Me scattering events contributions in the range of the second coordination shell, which frequently can be overlapped with other relatively strong single-scattering and/or multiple-scattering paths stemming from framework atoms. XANES is highly sensitive to the copper oxidation state and can easily differentiate between linear or square planar sites, but its sensitivity towards Cu–Cu spacing is significantly limited compared with atomic species in the first coordination sphere. ‘Longer’ energy intervals of XANES for ML predictions is often overlooked but highly important.

This work deals with the problem of structure refinement for a system containing light atoms in the first coordination shell and a 3*d* metal ion in the second. As a model case we select a binuclear copper complex with Schiff bases that may represent the coordination environment for copper-substituted zeolites and similar structures with multinuclear sites. These molecules exhibit also high biological activity (Arunadevi & Raman, 2020 ▸; Vlasenko *et al.*, 2020 ▸; Burlov *et al.*, 2018 ▸), while the Cu–Cu distance and nature of the bridge ligation affects the super exchange interaction between bridged ligands (Adhikary & Koner, 2010 ▸; Mei *et al.*, 2017 ▸). The use of a model compound opens a unique opportunity to compare results obtained within several approaches and single-crystal X-ray diffraction (XRD). In this work we omit the problem of ligand environment classification and construct the theoretical training sample for a ML algorithm by varying the Cu–O/N and Cu–Cu distances. We compare theoretical and experimental XANES spectra but select the proper *R*-factor in the *k*-space to equilibrate between contributions of the first coordination sphere and bridged metal scatterer. We show that the pure XANES region within the first 70 eV above the absorption edge has negligible sensitivity to the Cu–Cu distance variation. Staying within the XANES fit approach up to 170 eV, and by transferring the energy axis to the wavenumber we extend the capabilities of such a methodology. As a result, the distances obtained in the Fourier-transformed EXAFS (FT-EXAFS), single-crystal XRD and proposed XANES fit procedure agree with each other both for the first and second coordination shells.

## 2. Methods

### 2.1. Synthesis

Dimeric complexes of copper(II), Cu1, are synthesized during the interaction of solutions of equimolecular quantities of alcohol and copper acetate hydrate in ethanol (yield 65–75%). A hot solution of 200 mg (1 mmol) copper acetate hydrate in 16 ml of ethanol was added to a 1 mmol hot solution of ligand precursor [312 mg of (5-ethoxy-1-methylbenzimidazol-2-yl)(4-methoxyphenyl)methanol in 10 ml of ethanol], and left at an elevated temperature for 16 h. The obtained precipitates were filtered, washed with alcohol, and dried. We also utilized a complex with longer Cu–Cu bridged distance, Cu2. Further synthesis details and crystallographic characterization are provided in the supporting information (SI).

### 2.2. Experimental X-ray characterization

Cu *K*-edge X-ray absorption spectra were measured at the Structural Materials Science beamline (Chernyshov *et al.*, 2009 ▸) using the equipment of Kurchatov Synchrotron Radiation Source (Moscow, Russia). The storage ring, with an electron beam energy of 2.5 GeV and a current of 80–100 mA, was used as the source of radiation. All spectra were collected in transmission mode using a Si(220) channel-cut monochromator. Three ionization chambers monitored the intensity of the X-ray beam before and after the sample, and after the reference sample for energy calibration. Background subtraction, normalization, energy alignment and extraction of the χ(*k*) oscillatory function were performed using the *Athena* program of the *Demeter* package (Ravel & Newville, 2005*b* ▸; Newville, 2001 ▸). FT-EXAFS analysis using the *Artemis* program included calculation of theoretical amplitudes and phases by the *FEFF6* code (Zabinsky *et al.*, 1995 ▸) and fitting in the *R*-space of the Fourier-transformed data *k* ^2^-weighted χ(*k*) applying a Δ*k* Hanning window from 3 to 11.0 Å^−1^ with the width of the window slope d*k* = 1 Å^−1^. Further details of the fit are provided in the text.

### 2.3. ML methods

The training set for the ML method was calculated by means of the full multiple-scattering approach. Two popular computer programs, namely *FDMNES* (Zabinsky *et al.*, 1995 ▸) and *FEFF* (Rehr *et al.*, 2010 ▸), provide similar results and a user interface for such calculations. We selected the first one due to the availability of a free license. The calculation was performed inside a sphere of 6.0 Å radius around the absorbing Cu and unlimited expansion in spherical harmonics (Lmaxfree keyword). Theoretical spectra were further convoluted to account for the corehole lifetime broadening and instrumental energy resolution, and an arctangent function was used to model the energy dependence of the Lorentzian width. The convolution parameters were adjusted first for the reference spectra and demonstrate satisfactory agreement with experimental data [see Fig. S1 of SI]. In the extended energy region, the background spline is constructed with the *AUTOBK* function from the *Larch* package (Newville, 2013 ▸) with default parameters.

Quantitative analysis of the spectra was performed with the open-source *PyFitIt* tool (Martini *et al.*, 2020*a* ▸). *PyFitIt*-based ML methods can be applied in two different ways, depending on the choice of the input parameters and target function. The simplest approach is to use the training dataset to establish the spectrum → geometry correspondence. The ML algorithm takes the spectrum as input and predicts the structure geometry. This approach, called ‘direct’, suffers from ambiguity: different but symmetric structures correspond to the same spectrum. The regression models usually minimize the error value and thus predict the average structure, which is different from the correct symmetric ones. That is why we use the indirect approach, where ML algorithms are used for a fast spectrum approximation using structural parameters, *i.e.* for establishing the correspondence geometry → spectrum. The structural parameters are used as input and the algorithm predicts the spectrum quickly, replacing time-consuming calculations. The approximated spectra, for different structural parameters, are compared with the experimental one until the best agreement is obtained.

In that case we use the radial basis functions’ approximation as an ML method. It constructs a continuous approximation of a spectrum, μ(*E*), as a function of structural parameters **P** = (*p*_1_, *p*_2_,…, *p*_*k*_). The unknown function μ(*E*, **P**) is represented in terms of a set of radial basis functions characterized by polynomial terms,

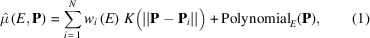

where *K*(*r*) is the radial basis function and Polynomial_*E*_(*P*) is a polynomial function of the *k*-dimensional vector of structural parameters *P* with energy-dependent coefficients. The quality of the approximation is evaluated with an *R* ^2^-score metric using the built-in function sklearn.metrics.r2_score(Y, predictedY, multioutput = YColumnWeights) where YColumnWeights are the differences between neighbor energy points. To compare the theoretical spectrum, theory(*x*), with the experimental one, exp(*x*), the *R*-factor is used,

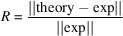

where

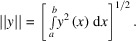

For each set of structural parameters *p*_1_,…, *p*_*k*_, the ML method predicts the theoretical spectrum, which is then compared with the experimental one. Thus we minimize the function of *n*-variables *R*-factor(*p*_1_,…, *p*_*k*_) in the region of the structural parameters variation. Here we compare fitting results of the two spectrum types: XANES on the energy interval 8970–9050 eV and EXAFS χ(*k*) on the interval *k* = 1.5–6.5 Å^−1^.

## 3. Results and discussion

**Figure 1:**
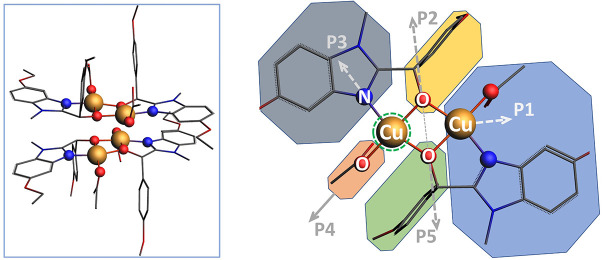
The whole structure of the studied copper complex (left) and splitting into fragments for deformations (right). The arrows show the direction of shift for each fragment.

Commonly in the fitting procedure the structural parameters are varied jointly with non-structural ones until a good agreement between calculated and experimental data is observed. Some of the non-structural parameters can be fixed in advance by using reference samples with known structure, that were measured under identical conditions. This procedure is well known in the field of Rietveld refinement or EXAFS analysis. When calculation of each spectrum takes a long time, as in XANES analysis, the spectra for different deformations can be calculated on discrete combinations of parameters first and then interpolation (Smolentsev & Soldatov, 2006 ▸) or an ML approximation (Martini *et al.*, 2020*a* ▸) is applied for continuous fit. In this work we rely on the radial basis function ML approximation. The molecule is divided into parts as shown in Fig. 1 ▸. Each part is associated with a functional group and shifted along the direction indicated by the arrows.

**Figure 2:**
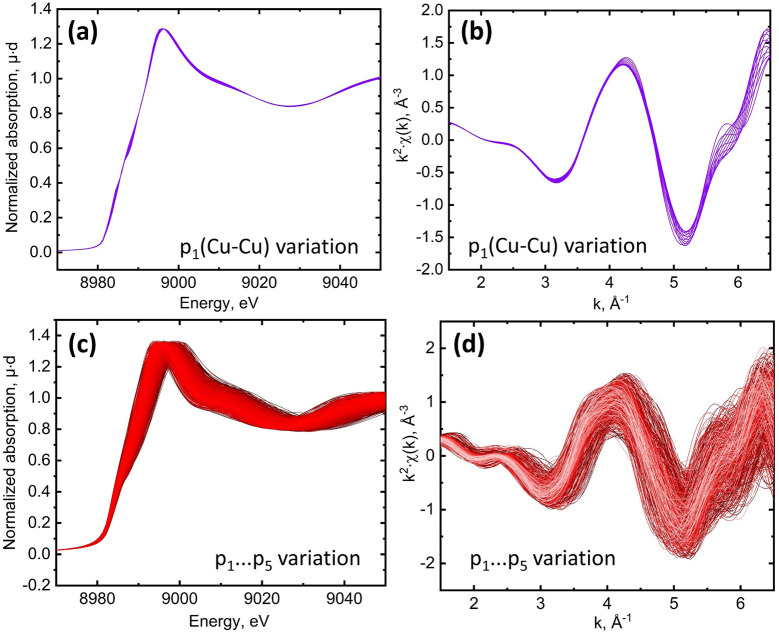
Sensitivity of spectra to structural deformations. Panels (*a*) and (*b*) show changes in μ(*E*) and χ(*k*) for varying *p*_1_ parameter (Cu–Cu distance only); panels (*c*) and (*d*) show spectral changes when all *p*_1_,…, *p*_5_ deformations are considered.

We shift simultaneously all parts using an improved hypercube sampling (IHS) scheme and Table 1 ▸ shows the ranges of varied parameters. IHS provides homogeneity of the sampled points in the multidimensional region of variation of structural parameters. For each set of deformations [*p*_1_,…, *p*_5_] we calculate the spectrum *ab initio* and store it in the training set that is finally composed of 1000 entries. Fig. 2 ▸ shows a series of calculated spectra when only the Cu–Cu distance is changed [parameter *p*_1_, panels (*a*) and (*b*)] and all distances are changed [parameters *p*_1_,…, *p*_5_, panels (*c*) and (*d*)]. Since structural parameters vary over large ranges, the smaller number of spectra in the training set was not enough for a good approximation. The radial basis functions (RBFs, ML algorithm) provide a reasonable quality of spectrum approximation both in the XANES region (*R* ^2^-score = 0.92) and for χ(*k*) (*R* ^2^-score = 0.96 for *k* = 1.5–6.5 Å^−1^). Fig. 2 ▸ contains the near-edge region within the first 70 eV above the absorption edge and that is strongly affected by multiple scattering and the more extended energy region of the spectrum. The common approaches based on the finite difference method (FDM) or full multiple scattering (FMS) can be straightforwardly applied for photoelectron energies up to 200 eV and even further with some modifications on the grid interpoint distance and number of spherical harmonics in the boundary sphere expansion (Bourke *et al.*, 2016 ▸).

To stay within the XANES fit methodology and without need of introducing new parameters from the EXAFS fit (Debye Waller and 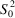 ) we limit the calculation up to the first 170 eV. Also we use a trick from EXAFS to increase the contribution from higher *k*-numbers. The spectrum is converted to *k*-space and multiplied by *k* ^2^. This balances the contributions from the near-edge region and the beginning of the higher *k*-region in the calculated *R*-factor.

The calculated sample of 1000 spectra after applying convolution turns out to have a stronger dependence on all the parameters, so determining the distance between the atoms of the copper is a difficult task. We further explore Fig. 2 ▸ and note that the near-edge region is not sensitive to the Cu–Cu distance when a metal atom is located in the bridged coordination. Only minor changes can be seen in the spectrum in Fig. 2 ▸(*a*). On the contrary this region is quite sensitive to the first coordination sphere as shown in Fig. 2 ▸(*c*). The white-line intensity and position of the pit are varied. We showed (Guda *et al.*, 2021 ▸) that the first pit is similarly important as positions of maxima and should not be overlooked. At the same time in the longer interval in the *k*-converted spectrum we observe a signal appearing from Cu–Cu scattering, as expected from the dependency of the scattering amplitude on atomic number (Martini *et al.*, 2020*b* ▸). Finally, Fig. 2 ▸(*d*) shows that both the near-edge and extended region strongly depend on the variation of distances in the first coordination shell.

The previous visual inspection of the training sample is an important step for further ML analysis. The quality of the prediction depends on the sensitivity and ambiguity of the spectral data on the structural parameters. If physical principles do not provide the required sensitivity, the expected quality of the prediction would be low. We noticed that the inclusion of a higher-energy interval in the XANES spectrum and converting into *k*-space along with multiplying by *k* ^2^ improves the sensitivity. We also note that while this information is well known in EXAFS fitting we still used a very short energy interval that is not sufficient for Fourier transform analysis. We continue with the next step and performed training. The set of calculated spectra was used to adjust coefficients in the RBF approximation [see equation (1)] and establish a relationship between deformations and spectral changes that substitute *ab initio* calculations and thus make it similar to Rietveld or EXAFS fitting.

**Figure 3:**
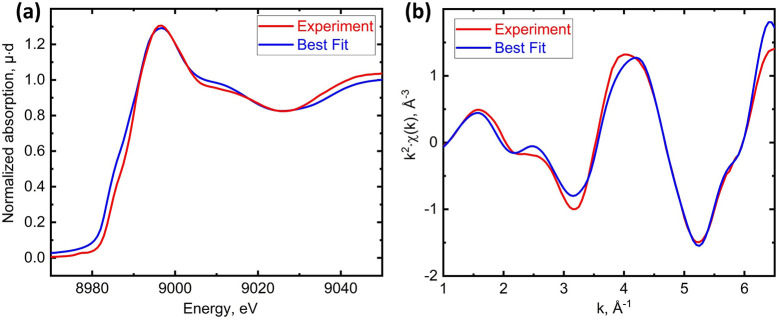
Best fits using the first 70 eV above the absorption edge (*a*) and 170 eV converted to *k* ^2^χ(*k*) (*b*).

The structural parameters are the input and an algorithm predicts the spectrum quickly, replacing time-consuming calculations. *PyFitIt* uses optimization algorithms to iteratively adjust the structure parameters to minimize the difference between the predicted spectrum and the experimental one. Fast spectrum approximation enables global minimum finding in reasonable time. Fig. 3 ▸ shows the best fits, *i.e.* calculated spectra for such structural parameters that provide minimal *R*-factor. Table 2 ▸ compares the structural parameters obtained from XANES, classical EXAFS and longer XANES fit.

Three independent methods provide good agreement with crystallographic data and indicate possible ambiguities. In the simplest case of an idealized coordination shell with equal distances, XAS reveals its high sensitivity to distance variations smaller than 0.01 Å. Difficulties appear when several different distances can be present in the same coordination shell thus increasing the number of variable parameters. Being most sensitive to the center of mass for distances, XANES has a much lower sensitivity to further distortions [see, for example, the quantitative analysis with principle component decomposition by Martini *et al.* (2021 ▸)]. We also showed this effect for the Fe(terpy)_2_ complex in the excited state [see Fig. 17 of Martini *et al.* (2020*b* ▸)]. Therefore, in Table 2 ▸ we report not the independent parameters *p*_1_,…, *p*_5_ but their average.

FT-EXAFS reproduces CIF data and the Cu–Cu signal is clearly visible in the wavelet-transform maps (Fig. S2 of SI). This approach is also superior for two different distances in the same coordination shell but still provides a difference with the CIF in the range of several hundredths of an angstrom. Many fits for short XANES provided a similar *R*-factor but different Cu–Cu distances. Since a short energy range has negligible sensitivity to the Cu–Cu distance [see Fig. 2 ▸(*a*)] we do not report the fitted value. The heat maps around the global minimum of the *R*-factor help to visualize both the correlations between structural parameters and the sensitivity of the chosen comparison metrics to the deformations.

**Figure 4:**
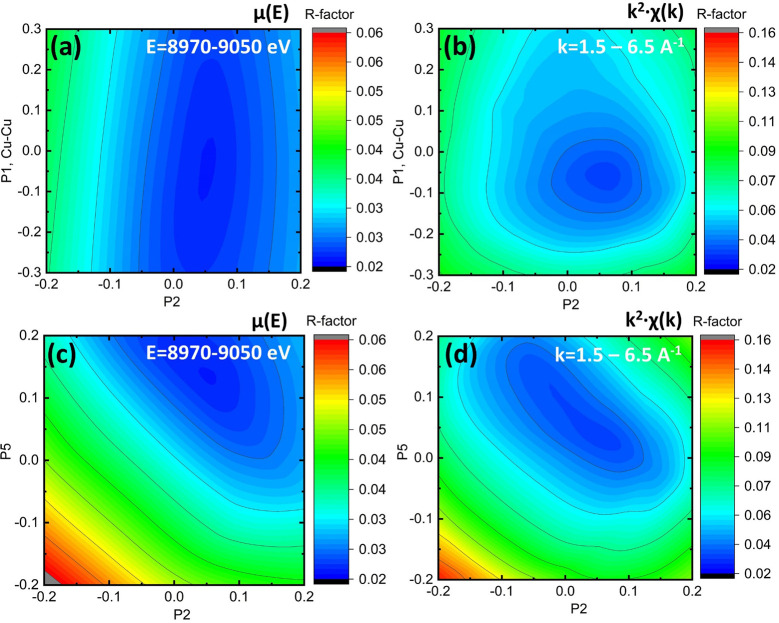
Variation of the *R*-factor in the proximity of the best fit. Panels (*a*) and (*c*) show the *R*-factor calculated for the XANES region (first 70 eV of the spectrum). Panels (*b*) and (*d*) show the *R*-factor calculated for the extended XANES region [first 170 eV and converted into *k* ^2^χ(*k*)]. The fit is shown in the space of two parameters: Cu–O and Cu–Cu bonds [panels (*a*) and (*b*)] or two distances in the first coordination shell [panels (*c*) and (*d*)].

Fig. 4 ▸ shows the variation of the *R*-factor when the Cu–Cu distance is varied simultaneously with the Cu–O distance in the first coordination shell. These maps demonstrate the relative sensitivity of the approach to different fitted parameters. It is clear from Fig. 4 ▸(*a*) that the short XANES region has lower sensitivity to the Cu–Cu bond distance as compared with *k* ^2^-weighted longer XANES in Fig. 4 ▸(*b*). The region of the global minimum in panel (*a*) has the shape of a valley elongated parallel to the *p*_1_ axis (Cu–Cu bond length). The sensitivity of the *R*-factor to the metal–metal bond distance is almost ten times smaller than to the nearest Cu–O bond length [compare the vertical and horizontal scales of the dark blue ellipse in Fig. 4 ▸(*a*) that indicate the region of a good fit]. The opposite situation is shown for the *k*-transformed longer XANES applied for the fit in Fig. 4(*b*). The region of best fit has now circular shape, thus implying equal sensitivity to both Cu–Cu and Cu–O distances.

Another ambiguity becomes clear upon inspection of Figs. 4 ▸(*c*) and 4 ▸(*d*), dedicated to the independent fit of the two Cu–O/N distances in the first coordination shell. The region of small *R*-factor is shaped in the valley parallel to the line *y* = −*x* + *b*. This line describes structures with constant average Cu–O/N distance in the first coordination shell. On the contrary, the *R*-factor varies quickly in the direction perpendicularly to this line, *i.e.* when the mean distance changes. These heat maps help researchers to understand the existing correlations between parameters in the structural fit and resulting ambiguities that are related to the intrinsic physical processes in XAS.

**Figure 5:**
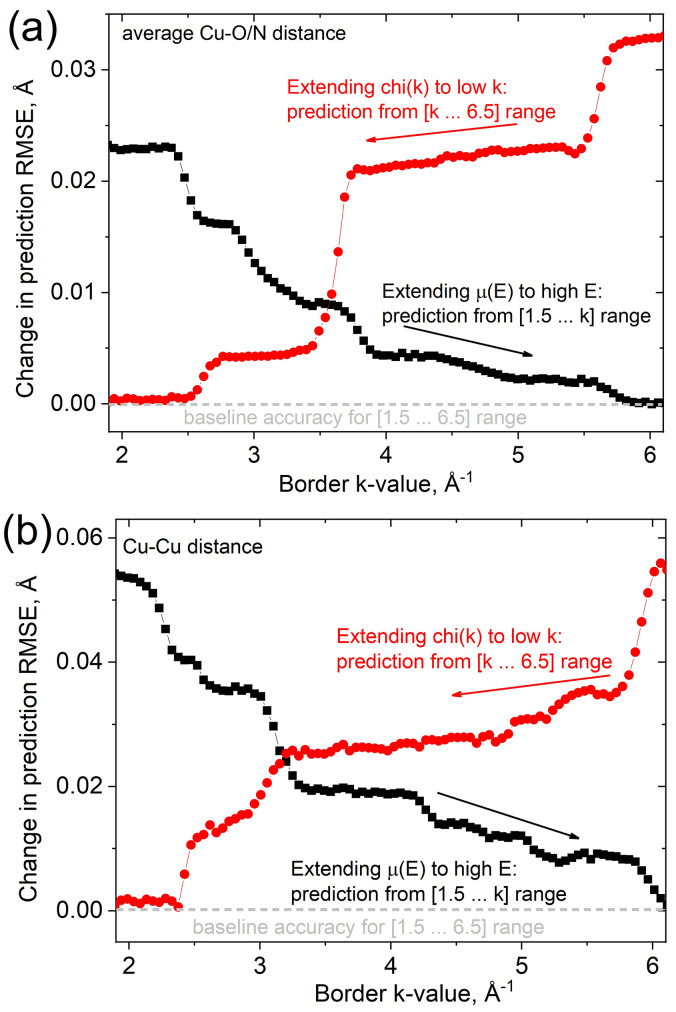
Estimation of the RMSE from the cross-validation scheme for average Cu–O/N (*a*) and Cu–Cu (*b*) distances by using a theoretical training set and limiting χ(*k*) to the [1.5…*k*] or [*k*…6.5] interval (relative to the baseline accuracy of the full range; closer to the baseline is better). Red curves show the variation of the uncertainty in the interatomic distance prediction when the left border of the interval is varied [meaning extension of χ(*k*) to lower *k*-values]. Black curves show the variation of the uncertainty when the right border of the interval is varied (*i.e.* extension of XANES to higher *k*-values).

The Cu–Cu distance is the parameter with the largest error in determination. The second copper atom is located at a longer distance and its scattering contribution is much smaller than that of the O/N atoms from the first coordination sphere, even despite larger Cu atomic mass. The dependence of scattering amplitude on atomic number suggests that the longer interval of the spectrum in *k*-space would be more sensitive to the Cu–Cu distance. We have applied a direct approach, which implies training an algorithm to predict the Cu–Cu distance directly based on the input spectrum. We varied the value for the right or left border of the spectrum interval used for prediction. The results for average Cu–O/N and Cu–Cu distances are shown in panels (*a*) and (*b*) of Fig. 5 ▸, respectively. For each range of wavenumbers, we have performed cross-validation based on a theoretical training set and report the correlation between root mean squared error (RMSE) and interval length. Red curves demonstrate the fit improvement when the EXAFS interval is expanded towards lower *k*-values. Black curves, on the contrary, address the uncertainty by extending the XANES fit interval towards higher energies. Both approaches are rarely applied in practice since multiple-scattering effects are important at low *k*-values and the EXAFS formula fails to describe this region. XANES calculations in turn become more CPU- and RAM-demanding at higher energies within finite difference of multiple-scattering approaches. However, with this work we would like to promote a moderate extension of the region for the XANES fit. The resulting interval, 1.5–6.5 Å^−1^ (∼170 eV), is optimal in terms of experimental registration (*operando* and time-resolved studies preventing good quality EXAFS acquisition) and computation (both FDM and FMS approaches work fast with slightly increased amount of spherical harmonics in the wavefunction expansion).

## 4. Challenges and future perspectives

**Figure 6:**
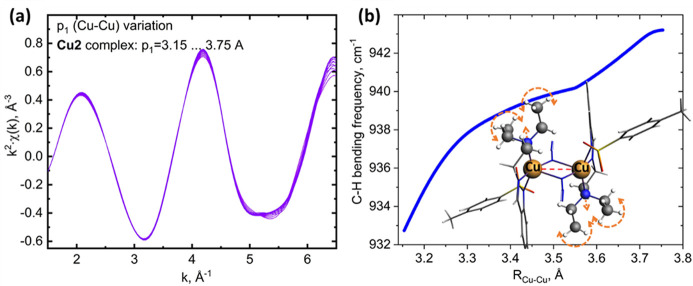
Spectral changes for a binuclear complex with longer equilibrium Cu–Cu distance. (*a*) Low sensitivity to the Cu–Cu distance of *k* ^2^-weighted χ(*k*) in the range 1.5–6.5 Å^−1^. (*b*) Variation of the C—H bending frequency of tri­ethyl­amine ligands as a function of Cu–Cu distance. The C—H bending mode is denoted with dashed orange arrows.

Some bridged metal–metal configurations show even longer bond lengths. In this section we address this for the second copper complex Cu2 with a crystallographic distance of 3.45 Å. A similar methodology in the short *k*-interval has a much lower sensitivity if compared with Cu1 [see Fig. 6 ▸(*a*)]. Since we face the physical limitation of the method itself the use of sophisticated ML algorithms may hardly help to address this problem. An alternative approach is using complementary techniques that may provide additional sensitivity to the metal–metal bond length. Infrared (IR) and Raman spectroscopies largely contributed to the active-site structure determination. Being a ubiquitous and convenient laboratory spectroscopy method, vibrational spectroscopy can be used to provide useful insights on the active centers including metal sites’ structure elucidation (Vanelderen *et al.*, 2015 ▸; Woertink *et al.*, 2009 ▸; Palagin *et al.*, 2021 ▸), including second sphere characterization (Snyder *et al.*, 2018 ▸), active sites formation (Ipek *et al.*, 2017 ▸) and metal sites structural variations, tracked *in situ* or *operando* (Groppo *et al.*, 2023 ▸). Recently, based on Raman spectroscopy supplied with density functional theory calculations, Snyder and co-coworkers (Snyder *et al.*, 2018 ▸) have discriminated between two [Cu_2_O]^2+^ motifs exhibiting very similar geometric and electronic structures but rather different CH_4_ activation enthalpy. Recent work by Palagin *et al.* (2021 ▸) reports mapping of different IR spectral regions to specific copper species as a function of zeolite topologies and Si:Al ratio as well as exploring the modifications in the stretching frequencies of typical probe molecules (such as CO, NO) depending on type and nuclearity of Cu sites.

We have performed a search in vibrational modes providing highest sensitivity to the Cu–Cu distance. The frequencies were calculated using the vibrational analysis module of the atomic simulation environment (ASE) (Larsen *et al.*, 2017 ▸) with the graph neural network with the three-body interactions universal interatomic potential (M3GNet) (Chen & Ong, 2022 ▸) as a calculator of forces and energies. The finite difference approximation of the Hessian matrix (the matrix of second-order derivatives of a potential energy with respect to the atomic coordinates) was diagonalized to compute the vibrational frequencies of each structure in the generated sample. Fig. 6 ▸(*b*) shows the frequency variation for the mode with largest sensitivity. The frequency depends monotonously on the Cu–Cu distance and falls in the wavenumber region that is accessible for most IR spectrometers.

The above-described ML methodology can be straightforwardly extended for simultaneous IR and XAS fits. The addition of FTIR data to the XAS fit would reduce ambiguity for prediction of intermetallic distance in bridged complexes. Presented ML methods will also contribute to IR and Raman spectroscopies for copper dimer structure investigation, as they contributed vibrational spectroscopies previously in broader cases (Lansford & Vlachos, 2020 ▸; Skorynina *et al.*, 2023 ▸).

## 5. Conclusions

Our work addresses improvement of the fit quality based on the near-edge region of the X-ray absorption spectrum. The XANES region is often used in the task of classification of the ligand environment, symmetry and metal oxidation state. The EXAFS region is more sensitive to coordination numbers and bond distances, but often *operando* studies complicate measurement of the high-quality EXAFS data needed for the Fourier transform analysis. Therefore, remaining within the methodology of XANES calculations (full multiple scattering or finite difference methods) we extend the calculation region up to ∼170 eV and convert the spectrum into the *k* ^2^χ(*k*) form. Such a transformation equilibrates two energy regions upon calculating the *R*-factor: the first ∼50 eV are sensitive mostly to the oxidation state and first coordination shell while being silent to the bridged metal–metal bond, and the extended region, up to 170 eV above the absorption edge (or *k* = 6.5 Å), that is already sensitive to metal–metal scattering.

The set of ‘long’ XANES spectra constituted training a sample for the RBF algorithm and ML methodology allowed us to estimate ambiguities during the fit procedure via cross-validation analysis and fast evaluation of the *R*-factor contour plots around the best fit. The practical application was demonstrated for a multinuclear copper complex where we achieved a good agreement with crystallographic data upon fitting distances in the first and second coordination spheres around the Cu atom. Considering the close similarity between binuclear molecular complexes investigated in this work and metal exchanged zeolites, the reported methodology might be readily extrapolated for a wide class of transition-metal-based single-site catalysts.

## 6. Related literature

The following references, not cited in the main body of the paper, have been cited in the supporting information: Battye *et al.* (2011 ▸); Chernova *et al.* (1971 ▸); Dolomanov *et al.* (2009 ▸); Evans (2006 ▸); Lazarenko *et al.* (2017 ▸); Lomakin *et al.* (1963 ▸); Sheldrick (2015 ▸); Vlasenko *et al.* (2021 ▸); Winn *et al.* (2011 ▸).

## Supplementary Material

Sections S1 to S6. DOI: 10.1107/S1600577524002091/ok5107sup1.pdf

## Figures and Tables

**Table 1:** Range of the variation of the structural parameters for the fit

	**P**1	**P**2	**P**3	**P**4	**P**5
Deformation range (Å)	−0.3 to +0.3	−0.2 to +0.2	−0.2 to +0.2	−0.2 to +0.2	−0.2 to +0.2
Initial value (Å)	2.97	1.95	1.93	1.92	1.90

**Table 2:** Refined distances (Å) and their uncertainties in the first coordination shell and bridged Cu–Cu bond distance The table compares crystallographic values, classical Fourier-transformed EXAFS in the range *k* = 3–11 Å^−1^, the XANES fit using the first 70 eV of the spectrum and the XANES fit in *k*-space (see Fig. S1 in SI for details of the classical FT-EXAFS fit).

Coordination sphere	CIF	FT-EXAFS	XANES *E* = 8970–9050 eV	XANES *k* = 1.5–6.5 Å^−1^
O	1.92	1.91 (0.01)	1.90 (0.03) (average 1.82 and 1.98)	1.89 (0.02) (average 1.86 and 1.92)
O	1.92			
N	1.95	1.99 (0.01)	2.00 (0.03) (average 1.98 and 2.02)	1.96 (0.02) (average 1.93 and 1.99)
O	1.95			
Cu	2.97	2.96 (0.03)	–	2.92 (0.06)
