# Supplementary material for: Improving sensitivity of XANES structural fit to the bridged metal–metal coordination
Source: J Synchrotron Radiat. 2024 Mar 26;31(Pt 3):447–55. doi: 10.1107/S1600577524002091 (PMC11075727; doi:10.1107/S1600577524002091)
Supplement: Supplementary file 1 [file s-31-00447-sup1.pdf]

Synchrotron JSR JOURNAL OF SYNCHROTRON RADIATION

**Volume 31 (2024)**

**Supporting information for article:**

# Improving sensitivity of XANES structural fit to the bridged metal–metal coordination

**S. V. Abrosimov, B. O. Protsenko, A. S. Mannaa, V. G. Vlasenko, S. A. Guda, I. A. Pankin, A. S. Burlov, Y. V. Koshchienko, A. A. Guda and A. V. Soldatov**

### S1. Adjusting convolution parameters for XANES spectra

The parameters of convolution (gamma hole, Elarge, Ecent, gamma max) were adjusted simultaneously for the series of copper complexes with known crystallographic structure. The criterium was the overall small R-factor between theoretical spectra calculated for CIF coordinates and experimental spectra for crystalline powders. Figure S1 shows comparison of theoretical spectra before and after convolution for the **Cu1** complex. In the extended region the arctangent convolution does not reproduce experimental broadening and further inclusion of Debye-Waller decay is needed. Therefore, we restrict the fit up to $6.5A^{-1}$ in the main text.

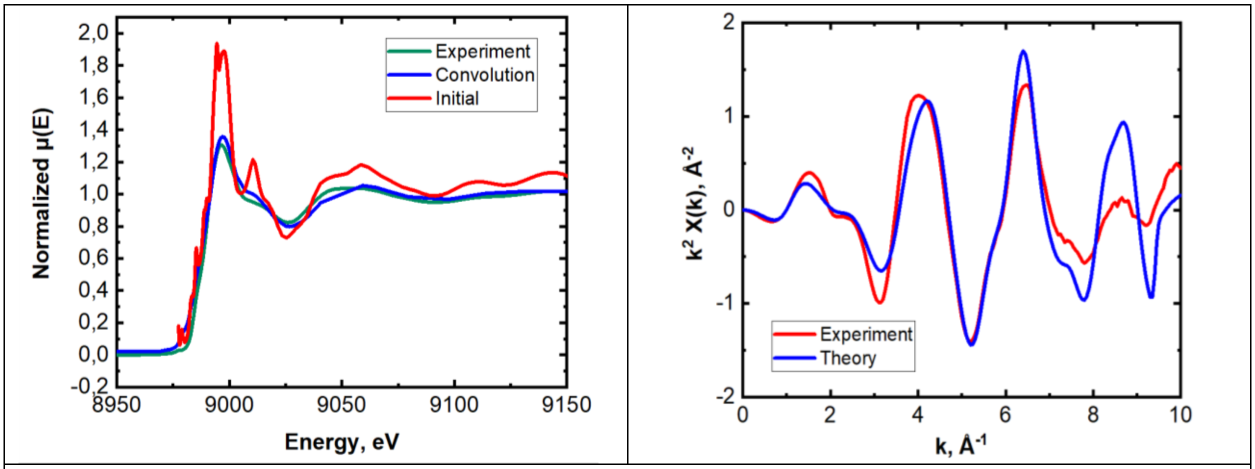


**Figure S1** Comparison between calculated spectra with and without Lorentzian convolution and experiment (left). Right panel shows the same spectra but for $k^2\chi(k)$ data.

### S2. Fourier Transform EXAFS fit

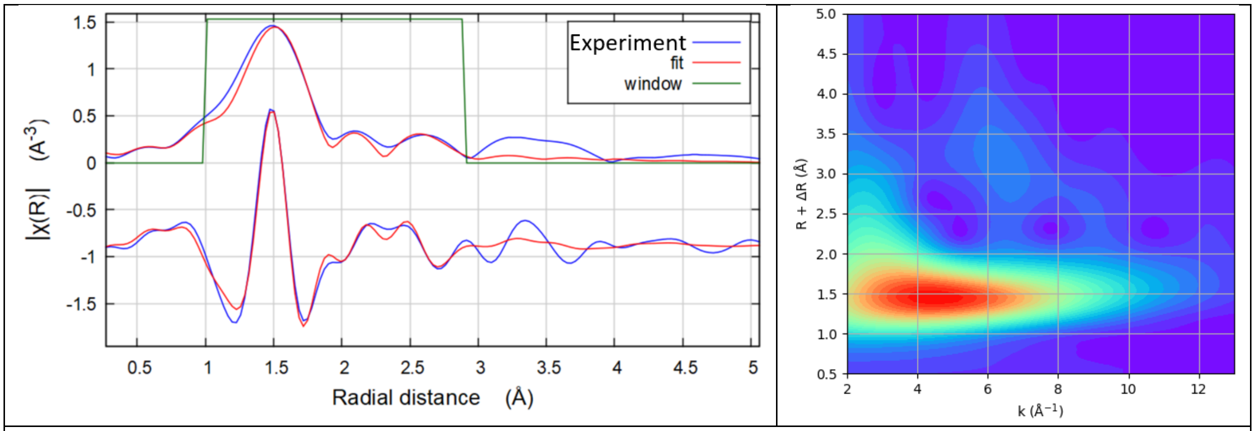


**Figure S2** Experimental magnitude and real part of the Fourier transformed signal plotted along with the best fit. Right panel shows wavelet transform of the χ(k) data, indicating Cu-Cu scattering contribution in the region of k=5.5 – 8 $Å^{-1}$ and R=2.5 – 3.5 Å.

**Table S1** Refined parameters of FT-EXAFS fit using the range [1…2.9] Å in the R-space and [3…11] $Å^{-1}$ in the k-space.

| Atom | CN | R, Å | $\sigma^2$, $Å^2$ | $E_0$, eV | $S_0^2$ | $\Re$, % |
|---|---|---|---|---|---|---|
| O/N | 2 | 1.91 (0.01) | 0.0025 | 5.1 (0.5) | 1 | 1.9 |
| O/N | 2 | 1.99 (0.01) | 0.0025 | | | |
| Cu | 1 | 2.96 (0.03) | 0.0080 | | | |

### S3. Details on X-ray absorption measurements

The Cu K-edge XAS spectra of copper complexes were measured at room temperature on the beamline "Structural Materials Science" [1] using the equipment of the Kurchatov Synchrotron Radiation Source (Moscow, Russia). The storage ring with an electron beam energy of 2.5 GeV and a current of 100–130 mA was used as the source of radiation. Si(111) channel-cut monochromator was used to scan the photon energy of the synchrotron X-rays in transmission mode. Three ionization chambers were used to monitor the intensity of the X-ray beam before and after the sample and after the reference copper foil for energy calibration purposes by taking the K-edge energy of copper metal as 8979 eV. Background subtraction, normalization of the data, energy alignment, and extraction of EXAFS data ($\chi_{exp}(k)$ oscillatory functions) were performed in the Athena program of the IFEFFIT package [2]. The radial pair distribution functions around the Cu ions were obtained by the Fourier transformation (FT) of the $k^2$-weighted EXAFS functions $\chi_{exp}(k)$ over the ranges of photoelectron wave numbers k =2.6–13.0 $Å^{-1}$. The Artemis program was employed to fit the theoretical model to the experimental data in R-space. The different fitting

parameters, including the interatomic distances ($R_i$), the relative mean-square displacement of the atoms from thermal motion and static disorder, also known as Debye-Waller factors ($\sigma^2$), zero energy shift ($\Delta E_0$), and passive electron reduction factor ($S_0^2$), were found by the non-linear fit of theoretical spectra to experimental ones. As a starting point for fitting, we used crystallographic coordinates obtained from single-crystal X-ray diffraction data of Cu complexes.

### S4. Details on crystallographic characterization

The single-crystal X-ray diffraction data for copper complexes were collected at the "Belok" beamline (Rayonix SX165 CCD detector, T = 100 K, $\lambda$ = 0.9699 Å) at the Kurchatov Synchrotron Radiation Source at NRC "Kurchatov Institute" (Moscow, Russia) [3]. In total, 720 frames were collected with an oscillation range of 1.0° in the $\varphi$ scanning mode using two different orientations for the crystals. The semi-empirical correction for absorption was applied using the CCP4 scaling program SCALA [4]. The data were indexed and integrated using the utility iMOSFLM from the CCP4 software suite [5]. The structures were solved by the intrinsic phasing modification of direct methods [6] and refined by a full-matrix least-squares technique on $F^2$ with anisotropic displacement parameters for all non-hydrogen atoms. The hydrogen atoms were placed in calculated positions and refined within the riding model with fixed isotropic displacement parameters [Uiso(H) = 1.5Ueq(C) for the methyl groups and 1.2Ueq(C) for the other groups]. All calculations were carried out using the SHELXTL program [7]. OLEX2 software suite [8] was used for some processing steps and structure visualization.

### S5. Synthesis and characterization of free ligands and its copper complexes

All solvents, copper acetate hydrate, butyllithium (1.6 M) in hexane, 4-methoxybenzaldehyde, *N*,*N*-diethylethylenediamine, sodium azide were purchased from Alfa Aesar and used as received. 5-Ethoxy-1-methylbenzimidazole [9] and 2-(*N*-tosylamino)benzaldehyde [10] have been synthesized using the reported procedures. The synthesis of complex **Cu1** was carried out according to the scheme 1.

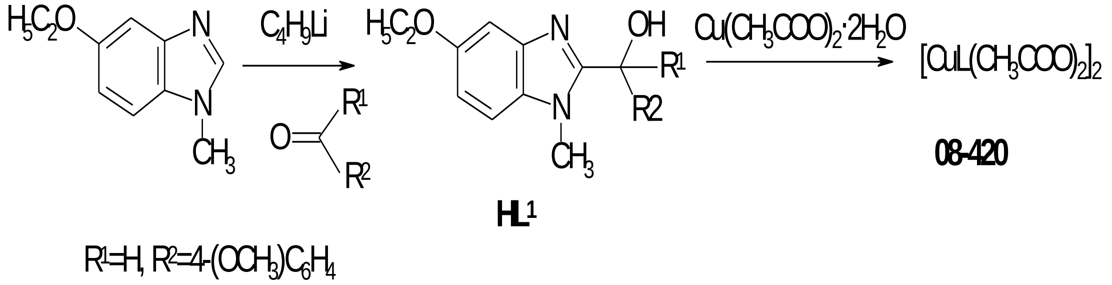


**Scheme 1** The synthesis of complex **Cu1**

A solution of 5-ethoxy-1-methylbenzimidazole (1.32 g, 7.5 mmol) in 8 mL $Et_2O$ at -10 °C in argon atmosphere was added to a solution of butyllithium (1.6 M) (4.7 mL, 7.5 mmol) in hexane. The mixture was refluxed for 1 h, then was added to a solution 4-methoxybenzaldehyde (1.02 g, 7.5 mmol) in 5 mL $Et_2O$. The mixture was stirred for 1 hour 30 minutes, 15 ml of 15% hydrochloric acid was added, and treated with a 22% aqueous ammonia solution until pH > 7.

The precipitate of 5-ethoxy-1-methylbenzimidazol-2-yl)(4-methoxyphenyl)methanol (**HL$^1$**) was filtered off, washed in water, and dried. Yield 2.01 g (78 %). Colorless crystals from ethyl acetate, m.p.177-178 °C. Anal. calc. for $C_{18}H_{20}N_2O_3$, %: C 69.21, H 6.45, N 8.97. Found, %: C 69.15, H 6.50, N 9.02. IR spectrum, selected bands, $cm^{-1}$: 3050 br.w. ν(OH). $^1$H NMR, δ(ppm) ($CDCl_3$): 1.45 (3H, t, $^3J$= 7.0 Hz, $CH_2\underline{CH_3}$), 3.45 (3H, s, $NCH_3$), 3.78 (3H, s, $OCH_3$), 4.07 (2H, $^3J$= 7.0 Hz, $\underline{CH_2}CH_3$), 4.97 (1H, br.s, OH), 5.96 (1H, s, $\underline{CH}$(OH)), 6.85 (2H, d, $^3J$= 8.7 Hz, $C_{Ar\text{-}H}$), 6.90 (1H, dd, $^3J$= 8.8 Hz, $^4J$= 2.3 Hz, $C_{Ar\text{-}H}$), 7.11 (1H, d, $^3J$= 8.8 Hz, $C_{Ar\text{-}H}$), 7.21 (2H, d, $^4J$= 2.3 Hz, $C_{Ar\text{-}H}$), 7.28 – 7.30 (1H, m, $C_{Ar\text{-}H}$).

A hot solution of 200 mg (1 mmol) copper acetate hydrate in 16 ml ethanol was added to a hot solution of 312 mg (1 mmol) HL in 10 ml ethanol and left to stand at room temperature for 16 hours. The precipitate of complex 08-420 was filtered off, washed in ethyl, and dried. Blue crystals, m.p. >250 ºC. Yield 282 mg (65 %). Anal. calc. for $C_{40}H_{44}Cu_2N_4O_{10}$, %: C55.36, H5.11, Cu14.64, N6.46. Found, %: C55.30, H5.19, Cu 14.73, N6.39. IR spectrum, selected bands, $cm^{-1}$: 1606 s, ν(C=O).

Synthesis of N-{2-[(2-diethylaminoethylimino)-methyl]-phenyl}-4-methyl-benzenesulfonamide **HL$^2$** and his copper complex **Cu2** was carried out as shown in Scheme 2.

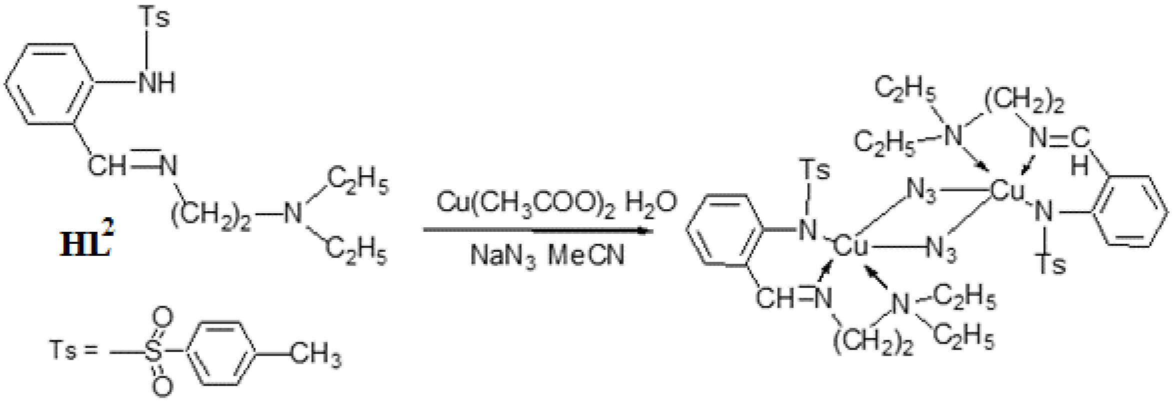


**Scheme 2** Synthesis of copper complex **Cu2**

The synthesis and molecular structure of the complex **Cu2** were described previously in [11].

#### S5.1. *N*-{2-[(2-Diethylaminoethylimino)methyl]phenyl}-4-methylbenzenesulfonamide (HL$^2$)

2-(*N*-Tosylamino)benzaldehyde (1.37 g, 5 mmol) was added to a solution of *N*,*N*-diethylethylenediamine (0.58 g, 5 mmol) in 20 mL of absolute benzene. The mixture was refluxed for 2 h with a Dean-Stark receiver until the water was completely separated. Benzene was distilled off using a rotary evaporator. The resulting oily product was recrystallized from 10 mL of methanol. Yield 1.55 g (85%). Light yellow powder, m.p. 44-45 °C. IR spectrum (powder), ν, $cm^{-1}$: 1633 s (CH=N), 1338 s, $\nu_{as}(SO_2)$, 1154 s, $\nu_s(SO_2)$. Anal. calc. for $C_{20}H_{27}N_3O_2S$, %: C 64.31, H 7.29, N 11.25. Found: C 64.28, H 7.32, N 11.20. $^1$H NMR (DMSO-$d_6$, 300 MHz), δ (ppm): 0.94 (6H, t, J = 7.2 Hz, 2 $CH_2\underline{CH_3}$), 2.29 (3H, s, C-$CH_3$), 2.53 (4H, q, J = 7.2 Hz, 2 $\underline{CH_2}CH_3$), 2.71 (2H, t, J = 6.3 Hz, $CH_2\underline{CH_2}N(C_2H_5)_2$), 3.68 (2H, t, J = 6.3 Hz, $\underline{CH_2}CH_2N(C_2H_5)_2$), 7.04 (1H, t, J = 7.5

Hz, $CH_{Ar}$), 7.30 (2H, d, J = 8.1 Hz, $CH_{Ar}$), 7.34 (1H, d, J = 7.5 Hz, $CH_{Ar}$), 7.45 (2H, t, J = 6.3 Hz, $CH_{Ar}$), 7.67 (2H, d, J = 8.1 Hz, $CH_{Ar}$), 8.45 (1H, s, CH=N).

### S5.2. Bis[(*N*-{2-[(2-diethylamino-ethylimino)-methyl]-phenyl}-4-methyl-benzenesulfonamido)] $Cu_2(\mu_{1,1}-N_3)_2$•2($CH_3CN$) CuB753

A solution of *N*,*N*-diethylethylenediamine (0.23 g, 2 mmol) in 5 mL of acetonitrile and a solution of sodium azide (0.13 g, 2 mmol) in 5 mL of acetonitrile were added to a solution of 2-(*N*-tosylamino)benzaldehyde (0.55 g, 2 mmol) in 15 mL of acetonitrile. The ligand was not isolated and used directly for the synthesis of copper complex **3**. A solution of copper(II) acetate monohydrate (0.4 g, 1 mmol) in acetonitrile (20 mL) was added to the solution of the ligand in acetonitrile (mL). The mixture was stirred at r. t. for 2 h. The precipitate of complex **3** was filtered off, washed twice with 5 ml of acetonitrile, and dried in a vacuum at 100 °C. Yield 0.85 g (82%). Green crystals, m.p. 218-219 °C. Anal. calc. for $C_{44}H_{58}N_{14}O_4S_2Cu_2$: C 50.90; H 5.63; N 18.89; Cu 12.24. Found: C 51.10; H 5.70; N 18.95; Cu 12.37 %. IR spectrum (powder), ν, $cm^{-1}$: 2058 vs ($N_3$), 1644 vs (CH=N), 1283 s, $\nu_{as}(SO_2)$, 1136 s, $\nu_s(SO_2)$.

*XRD data* - Crystallographic data for **Cu2** have been deposited with the Cambridge Crystallographic Data Center, CCDC 2064264. The supplementary crystallographic data can be obtained free of charge from the Cambridge Crystallographic Data Centre via www.ccdc.cam.ac.uk/data_request/cif.

### S6. Elemental analysis of synthesized sample

C, H, N elemental analyses were carried out using a Carlo Erba TCM 480 apparatus using sulfanilamide as a reference. The metal content was determined gravimetrically in the analytical laboratory of the Institute of Physical and Organic Chemistry (SFedU, Rostov-on-Don, Russia). Melting points were measured on a Kofler table. Infrared spectra were recorded using a Varian Excalibur-3100 FT-IR spectrophotometer for powdered compounds in the attenuated total reflectance (ATR) mode. $^1$H spectra were measured using a Varian Unity-300 (300 MHz) and Varian Unity-600 (600 MHz) spectrometers at ambient temperature in $CDCl_3$. The chemical shifts (δ) were referenced to residual solvent shifts in the respective deuterated solvents.

## References

[1] A.A. Chernyshov, A.A. Veligzhanin, Y.V. Zubavichus, Nucl. Instr. Meth. Phys. Res. A -Accelerators, Spectrometers, Detectors and Associated Equipment, 603 (2009) 95-98. DOI:10.1016/j.nima.2008.12.167.

[2] B. Ravel, M. Newville, J. Synchr. Rad. 12 (2005) 537-541. https://doi.org/10.1107/S0909049505012719.

[3] V. A. Lazarenko, P. V. Dorovatovskii, Y. V. Zubavichus, A. S. Burlov, Y. V. Koshchienko, V. G. Vlasenko, V. N. Khrustalev, Crystals 7(11) (2017) 1-19. DOI: 10.3390/cryst7110325.

[4] P. Evans, Acta Crystallogr. D62 (2006) 72-82. https://doi.org/10.1107/S0907444905036693.

[5] T.G.G. Battye, L. Kontogiannis, O. Johnson, H.R. Powell, A.G. W. Leslie, Acta Crystallogr. D67 (2011) 271-281. DOI: 10.1107 / S0907444910048675, M.D. Winn, C.C. Ballard, K.D. Cowtan, E.J. Dodson, P. Emsley, P.R. Evans, R.M. Keegan, E.B. Krissinel, A.G.W. Leslie, A. McCoy, S.J. McNicholas, G.N. Murshudov, N.S. Pannu, E.A. Potterton, H.R. Powell, R.J. Read, A. Vagin, K.S. Wilson, Acta Crystallogr. D67 (2011) 235-242. DOI:10.1107 / S0907444910045749.

[6] G.M. Sheldrick, Acta Crystallogr. A71 (2015) 3-8. doi:10.1107/S2053273314026370.

[7] G.M. Sheldrick, Acta Crystallogr. A71 (2015) 3-8. doi:10.1107/S2053273314026370.

[8] O.V. Dolomanov, L.J. Bourhis, R.J. Gildea, J.A.K. Howard, H. Puschmann, J. Appl. Cryst. 42 (2009) 339-341. https://doi.org/10.1107/S0021889808042726.

[9] A.N. Lomakin, A.M. Simonov, V.A. Chigrina, Zh. Obshchei Khim. 1963, 33, 204.

[10] N.I. Chernova, Y.S. Ryabokobylko, V.G. Brudz, B.M. Bolotin, Zh. Org. Khim. 7 (1971) 1680-1687.

[11] Valery G. Vlasenko, Anatolii S. Burlov, Yurii V. Koshchienko, Alexandra A. Kolodina, Yan V. Zubavichus, Victor N. Khrustalev, T. N. Danilenko, Alexander A. Zubenko, Leonid N. Fetisov, Alexander I. Klimenko. Inorganica Chimica Acta, 523 (2021) 120408. https://doi.org/10.1016/j.ica.2021.120408.
